# Effects of Tipranavir, Darunavir, and Ritonavir on Platelet Function, Coagulation, and Fibrinolysis in Healthy Volunteers

**DOI:** 10.2174/157016211796320306

**Published:** 2011-06

**Authors:** Jens J Kort, Stella Aslanyan, Joseph Scherer, John P Sabo, Veronika Kohlbrenner, Patrick Robinson

**Affiliations:** Boehringer Ingelheim Pharmaceuticals, Inc., Ridgefield, CT, USA

**Keywords:** Aggregation, aspirin, bleeding time, coagulation, fibrinolysis, platelet, protease inhibitor.

## Abstract

The use of HIV protease inhibitors (PIs) as part of antiretroviral therapy in the treatment of HIV-1 infection may be associated with an increased risk of bleeding. This prospective, randomized, open-label trial in healthy volunteers compared the effects of tipranavir/ritonavir (TPV/r), darunavir/ritonavir (DRV/r), and ritonavir (RTV) alone on platelet aggregation after a single dose and at steady-state concentrations. Subjects were selected on the basis of normal platelet aggregation and arachidonic acid (AA)-induced platelet aggregation inhibition after administration of a single 325-mg dose of aspirin. All 3 PI therapies were administered twice daily for 10 days.

In some but not all subjects, TPV/r inhibited AA-induced platelet aggregation and prolonged PFA-100^®^ closure time with collagen-epinephrine cartridge, which was of lesser magnitude and consistency compared with aspirin, but greater when compared to DRV/r and RTV. At least 2 subjects in each treatment arm showed complete inhibition of AA-induced platelet aggregation on treatment, and the magnitude of change in all platelet-function tests did not correlate with PI plasma concentrations. Effects of TPV/r on platelet aggregation were reversed 24 hours after the last TPV/r dose. None of the PI treatments tested were associated with increases in bleeding time, decreases in plasma coagulation factors, or increase in fibrinolysis. There was large inter-patient variability in antiplatelet effect for all PI treatments, ranging from no effect to complete inhibition of AA-induced platelet aggregation.

## INTRODUCTION

Long-term combination antiretroviral therapy (cART) is associated with various complications, including cardiovascular events [1], lipodystrophy [2], and non-AIDS-defining cancers [3].

Tipranavir (TPV), coadministered with low-dose ritonavir (TPV/r), is indicated for cART of HIV-1 infected patients who are treatment-experienced and infected with HIV-1 strains resistant to more than 1 protease inhibitor (PI) [4]. Rare bleeding events, including fatal and nonfatal intracranial hemorrhage (ICH) have been reported in HIV-infected patients receiving TPV/r or other PIs as part of cART [5-8], and an increased incidence of bleeding events in HIV-positive hemophiliacs treated with PIs has been observed [7,8]. The incidence of ICH events in TPV/r-treated patients is low, 0.26/100 person-years of exposure, or 13 cases of ICH in 6,840 patients in TPV/r pre-approval trials [6], and this incidence for ICH is similar to that of HIV-infected patients who received a non-TPV/r-containing cART based on retrospective analyses of 2 large patient cohorts (*N* > 78,000 HIV-infected patients combined) from the California state Medicaid program (Medi-Cal) and the U.S. Veterans Healthcare System (VA) [9,10].

The mechanism by which TPV or other PIs may increase the risk of bleeding is not known. Results from a retrospective analysis of plasma samples from patients in the RESIST studies showed that neither TPV/r nor comparator PI/r treatments produced decreases in levels or activity of vitamin K-dependent coagulation factors, factor V, and prolongation of prothrombin time (PT) and activated partial thromboplastin time (aPTT) [11]. In a recent study with platelet-rich plasma (PRP) taken from 5 HIV-infected patients receiving TPV/r-containing cART, inhibition of collagen- (p<0.001 at 4 hours after dosing) and adenosine diphosphate (ADP) (non- statistically significant)-stimulated platelet aggregation was observed [12].

The current study was undertaken to prospectively examine the effects of TPV/r on platelet function and plasma biomarkers of coagulation and fibrinolysis in healthy volunteers and to compare these effects with those of ritonavir (RTV) and darunavir (DRV) coadministered with RTV (DVR/r). DRV was selected as a comparator PI because, similar to TPV, it is coadministered with RTV and also indicated and frequently used for the treatment of HIV-1 infection in antiretroviral treatment-experienced adult patients infected with HIV-1 strains resistant to more than 1 PI.

## METHODS

### Study Design and Treatments

This was a prospective, open-label, randomized, and controlled single-center trial in healthy volunteers, in which 36 subjects were initially randomized 1:1:1 to treatment with TPV/r, DRV/r, or RTV. Due to 7 early discontinuations in the TPV/r arm, 11 additional subjects were allocated to the TPV/r group.

Following informed consent, subjects were screened at visit 1, baseline platelet-function tests were obtained for each eligible subject at visit 2, and at visit 3, each subject received a single dose of aspirin 325 mg followed by determination of platelet function after 4 hours. After a washout period of ≥ 14 days, subjects were randomized to 1 of 3 treatment groups. Group 1 received TPV/r 500 mg/200 mg, group 2 received DRV/r 600 mg/100 mg, and group 3 received RTV 100 mg monotherapy, each administered twice daily for 10 days. Pharmacodynamic (PD) biomarkers were assessed prior to PI dosing, after 1 day of dosing (day 1), after the last morning dose on day 10 (steady state), and 1 and 2 days after the last PI dose. Subjects were confined to the study unit for 2 overnight stays for aspirin dosing, and for an additional 13 overnight stays for the randomized treatment and study assessments.

### Study Subjects

The study protocol and related documents were approved by the institutional review board. Healthy men and women between 18 and 50 years of age who met all inclusion criteria (see Table **1**) were eligible to participate in this study. Of the 280 subjects screened, 52 met study inclusion criteria prior to receiving a single 325-mg dose of aspirin. Subjects were excluded from the study if they used an investigational agent within 30 days prior to visit 2; donated blood or plasma within 30 days prior to visit 2; used aspirin or any nonsteroidal anti-inflammatory drug, cyclooxygenase-2 (COX-2) inhibitors, dipyridamole, clopidogrel, ticlopidine, or other antiplatelet drug prior to visit 2; had peptic ulceration or a history thereof; had an active bleeding disorder or history thereof; used any over-the-counter medication within 7 days prior to visit 2 or were currently receiving any prescription drug; used proton pump inhibitors within 14 days prior to visit 2; or had vitamin E intake > 60 mg/day within 30 days prior to visit 2 or during the study. Women were excluded if they were pregnant, breastfeeding, not willing to use a barrier method of contraception from screening through the end of study, or were taking any hormonal therapy. The subjects were specifically instructed not to use any nonprescription medication during the study.

### Study Objectives

The primary objective of this study was to determine the effect of steady-state plasma concentrations of TPV/r on platelet-function tests and biomarkers of coagulation and fibrinolysis. The secondary objective was to compare the effects of TPV/r, DRV/r, and RTV on these markers. Additional comparisons were made for effects on platelet functions for single-dose aspirin versus TPV/r in individual subjects.

### Platelet-Function Tests

Platelet aggregation tests used light transmission aggregometry. Blood samples from healthy volunteers were collected from antecubital veins into vacutainer tubes containing citrate. Platelet-rich plasma was prepared immediately by centrifugation at 1200×g, and platelet aggregation studies were performed within 1 hour in the Hematology and Coagulation Laboratories at Johns Hopkins University using a Chrono-log Whole Blood Lumi-Aggregometer^®^ (model 560-Ca) as previously described [13]. Platelet aggregation was stimulated with 0.5 mM arachidonic acid (AA), 5 µM ADP, and 5 µg/ml collagen, and expressed as the percentage of light transmittance change from baseline using platelet-poor plasma as a reference at the end of recording time. Platelet aggregation curves were analyzed according to internationally established standards using Aggrolink^®^ software (unless otherwise stated, all equipment and reagents from Chronolog Corp., Havertown, PA).

Closure time (CT) of platelet-rich plasma was determined in a PFA-100^®^ platelet-function analyzer (Dade Behring, Miami, FL) using collagen/epinephrine (CEPI) and collagen/ADP standard test cartridges [13]. Bleeding time was measured using the Surgicutt^®^ fully automated incision-making device for bleeding time determination (ITC, Edison, NJ), and package instructions were followed by personnel performing the tests. A small incision was made with the instrument on the subject’s forearm, and blood was gently blotted every 30 seconds, without touching the incision. The bleeding time was reported out to the nearest 30 seconds.

Urinary thromboxane B_2 _(TXB_2_) metabolites were measured at Covance Laboratories using TXB_2_ Express Enzyme Immunoassay Monoclonal Antibody (Cayman Chemical Company, Ann Arbor, MI).

### Markers of Coagulation and Fibrinolysis

Prothrombin time; aPTT; fibrinogen; von Willebrand antigen; anti-thrombin III antigen/activity; factors II, VII, IX, and X activity; plasminogen activity; α-2 antiplasmin; D-dimer; and plasminogen activator inhibitor (PAI-1) were measured at Covance Laboratories (Indianapolis, IN).

### Plasma Concentrations of Tipranavir, Darunavir, and Ritonavir

Blood samples for determination of concentrations of TPV, DRV, and RTV in plasma were collected in 5-ml vacutainer collection tubes containing ethylene diamino tetraacetic acid (EDTA). Trough plasma samples for the PIs were collected prior to the morning dose on days 1, 2, 3, and 6, and at day 10 (steady state) after initiation of treatment. Steady-state pharmacokinetic (PK) profiles included 6 timed samples within 12 hours of the morning dose of PI on day 10. Post last-dose plasma samples were collected 24 hours and 48 hours after the last dose.

Plasma concentrations of TPV, DRV, and RTV were measured using validated and quality-controlled liquid chromatography coupled with tandem mass spectrometry (LC/MS/MS) performed by BASi Analytical Laboratories (West Lafayette, IN).

### Endpoints

The primary PD endpoint was the inhibition of platelet aggregation by AA, measured as area under the curve (AUC) at steady state (day 10), expressed as percent reduction from baseline. Secondary endpoints included change from baseline at days 1, 10, 11 (i.e., 1 day post treatment), and 12 (2 days post treatment) for 3 sets of biomarkers: (1) platelet function biomarkers: platelet aggregation in response to AA, ADP, and collagen; PFA-100 test with CEPI reported as CT in seconds; PFA-100 test with collagen/ADP reported as CT in seconds; urinary TXB_2_ metabolites; and bleeding time; (2) coagulation biomarkers: aPTT, prothrombin time, fibrinogen, von Willebrand and antithrombin III antigens and activity; and coagulation factors II, VII, IX, and X; and (3) fibrinolytic biomarkers: plasminogen activity, α-2 antiplasmin, D-dimer, and PAI-1. Effects of the single dose of aspirin on markers of platelet aggregation were determined at 4 hours after aspirin dosing (visit 3). The PI effects were compared with the aspirin effects as day 1 PI change versus aspirin change and day 10 PI change versus aspirin change.

PK parameters measured for exploration of the PD/PK relationship included maximum drug concentration (*C*_max_)_,_ minimum drug concentration (*C*_min_), and AUC_0-12_ for the 3 PIs (TPV, DRV, and RTV).

### Safety Assessment

Safety endpoints included treatment-emergent adverse events (AEs) and laboratory abnormalities. Standard safety reporting methods were used in this study.

### Statistical Methods

The primary analysis consisted of computing the 95% confidence interval (CI) for the percent reduction from baseline to steady state in AA AUC for TPV/r subjects, based on a normal distribution. The bounds of this CI were compared to a 50% reduction. The 50% reduction was empirically chosen to distinguish between subjects with clear changes in laboratory markers of aggregation and those with borderline or no effect. These analyses were also repeated for other time points as well as in DRV/r- and RTV-treated subjects. In addition, percentage of subjects with ≥ 50% reduction in AA AUC was tabulated and differences between the groups were analyzed using a Fisher’s exact test. Secondary analyses consisted of summary statistics of all biomarkers at all time points. Using 2-sided *t*-tests, changes in markers of platelet aggregation, coagulation, and fibrinolysis from baseline to steady state were compared in subjects receiving TPV/r versus comparator PI treatments. Linear regression analyses were performed to explore the relationships between all studied biomarkers and PK parameters (*C*_max_, AUC_0-12_, *C*_min_ at steady state, and *C*_min_ at day 1 of treatment and 24 hours and 48 hours after end of treatment). For select PD markers, comparisons were made with PK parameters to explore whether differences in TPV levels accounted for differences observed in their platelet-function tests.

Effects on markers of platelet aggregation observed in the different PI-treatment groups were compared with the effects of single-dose aspirin in the same subject, using a Wilcoxon-Mann-Whitney test.

The number of patients in each arm was calculated in order to have enough power to show that the 95% CI for the mean percent reduction from baseline in AA AUC does not include a 50% reduction. Assuming a mean AA AUC of 200 Vs at baseline and a standard deviation (SD) of 20 (both conservatively estimated from Van Ryn *et al*. 2004 [14], and an absolute mean reduction of 80 Vs (i.e., a 40% reduction in AA AUC) at steady state, 12 subjects would provide 88% power to conclude that the reduction in AA AUC was not deemed relevant (i.e., CI will not include a 50% reduction).

## RESULTS

### Subject Disposition and Demographic Characteristics

Subject disposition is shown in Fig. (**1**). Of 280 subjects screened, 52 received aspirin at visit 3; 47 subjects received at least 1 dose of PI and 5 subjects received aspirin but no other study medication. In all, 36 subjects were randomized to TPV/r (*n* = 12), DRV/r (*n* = 12), or RTV (*n* = 12); 11 subjects were allocated to TPV/r as replacements for 7 subjects who discontinued early in the TPV/r arm. In response to AA, 45 of 47 subjects demonstrated a return to normal in platelet aggregation after aspirin administration and following a washout period, and were considered evaluable. Overall, demographic characteristics were well matched between treatment arms (Table **1**). Of the 47 treated subjects, 60% were men, 75% were black, the mean age was 33 years, and 23% had a body mass index (BMI) ≥ 30 kg/m^2^ indicating obesity.

### Light Transmission Aggregometry Platelet Aggregation Tests

AA-induced platelet aggregation in response to a single dose of aspirin and over the course of treatment with TPV/r, DRV/r, and RTV is shown in Fig. (**2A**). AA-induced platelet aggregation was completely inhibited by aspirin in all subjects in each PI-treatment group, whereas the effect of each of the PI treatments was highly variable at each on-treatment time point. For the primary analysis, TPV/r at steady-state concentrations lowered AA-induced platelet aggregation by a mean of 43% (SD, 57.5; 95% CI, 12.6-73.9; Fig. (**2B**); this effect was significantly different compared with subjects treated with DRV/r but not versus subjects in the RTV-treatment group. At steady state, 43.8% (7/16) of subjects in the TPV/r group had ≥ 50% inhibition of platelet aggregation in response to AA versus 8.3% (1/12) in the DRV/r arm and 16.7% (2/12) in the RTV arm (Fisher exact p-values of 0.0882 and 0.2232 for comparing TPV/r to DRV/r and RTV, respectively). Inhibition of platelet aggregation was reversed in all subjects 24 hours after the last dose of PI treatment. AA-induced platelet aggregation of ≥ 50% inhibition was observed in a larger proportion of subjects (66.7% [14/21]) after 1 day of TPV/r treatment compared with steady state. The day 1 proportions of TPV/r patients with AA-induced platelet aggregation were significantly higher than DRV/r (25.0% [3/12]; p=0.0324) and RTV (8.3% [1/12]; p=0.0028).

**Fig. (3):**
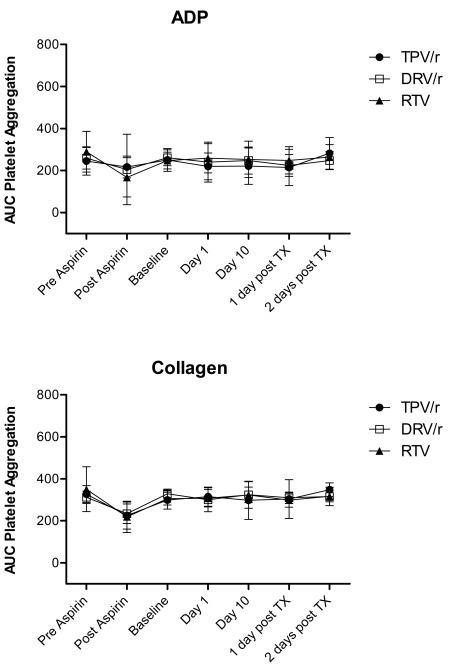
**Platelet aggregation in response to ADP and collagen over time (mean AUC ± SD [AUC unit])**. TX = treatment.

Treatment with TPV/r, DRV/r, and RTV did not have an effect on platelet aggregation in response to ADP and collagen (Fig. **3**). Only treatment with aspirin inhibited collagen-induced platelet aggregation significantly from baseline, and reduced collagen-induced platelet aggregation below reference range in 49% (22/45) of all subjects in the 3 PI-treatment groups combined.

### Other Platelet Function Tests

Mean values for other platelet function tests at various time points for subjects treated with TPV/r are shown in Table **2**. TPV/r had no significant effect on bleeding time or PFA-100 collagen/ADP CT. The PFA-100 CEPI CT was increased above the reference range (94-193 seconds) in 38% (8/21) and 25% (4/16) of TPV/r-treated subjects on day 1 and day 10 after dosing, respectively. Prolongation of the CEPI CT was reversed to normal values 24 hours after the last TPV/r dose in all subjects in that group. A total of 7 of 14 and 5 of 7 TPV/r-treated subjects with ≥ 50% inhibition of platelet aggregation in response to AA on day 1 and day 10, respectively, had no increase in PFA-100 CEPI CT above the normal reference range at the same time points. Thus, there was only partial overlap in effects of TPV/r on AA-induced platelet aggregation and CEPI CT. Urinary TXB_2_ metabolites in the TPV/r group were significantly increased from baseline after 10 days of treatment and remained elevated 48 hours after the last TPV/r dose.

In a comparison of treatment arms, PFA-100 CEPI CT (mean change ± SD in seconds) in the TPV/r group (+92 ± 73) was the only secondary platelet function marker with a change from baseline that was significantly and meaningfully different compared with DRV/r (-3.4 ± 15.2; p=0.0001) and RTV (-11.2 ± 19.9; p<0.0001) on day 1 of dosing. However, the mean (± SD) CEPI CT for TPV/r subjects did not change substantially above the upper limit of the reference range after 1 day of TPV/r dosing (193.1 ± 92 seconds) (Table **2**). The prolongation of the PFA-100 CEPI CT (mean increase ± SD) was significantly greater with aspirin (+124 ± 71 seconds) than with steady-state TPV/r (+56 ± 55 seconds; p=0.0135); mean ± SD absolute CEPI CT at steady-state TPV/r was 161.7 ± 56 seconds. Single-dose aspirin prolonged CEPI CT in 81% (17/21) of TPV/r subjects to above reference range (mean ± SD CEPI CT after single-dose aspirin, 249 ± 69 seconds).

A summary of within-subject comparisons of effects at steady state with aspirin versus TPV/r for the 4 selected platelet aggregation markers is shown in Fig. (**4**). With the exception of the absence of significant effects of aspirin and TPV/r on ADP-induced platelet aggregation, the magnitude of inhibition of AA- and collagen-induced platelet aggregation and prolongation of CEPI CT was significantly greater following a single dose of aspirin when compared with the effect of TPV/r at steady-state levels.

### Markers of Coagulation and Fibrinolysis

Changes of mean values for coagulation and fibrinolysis biomarkers at various time points for subjects treated with TPV/r are shown in Table **3**. Due to the multitude of tests being conducted without any adjustment for multiplicity, these results should be interpreted with caution as the probability of a chance finding with so many tests is high. Certainly, causal relationships cannot be inferred until the results are confirmed in another study.

There were statistically significant elevations from baseline to day 1 observed in plasminogen activity and PAI activity. Statistically significant changes from baseline to day 10 were observed in most of the coagulation and fibrinolysis biomarkers studied (12 out of 14 parameters) and these changes were mostly maintained until 48 hours after administration of the last dose of study drug. Only 3 markers were changed in the direction that could potentially decrease coagulation: antithrombin III antigen (increase from baseline mean of 104.29% to steady state mean of 134.44%), antithrombin III activity (increase from baseline mean of 100.57% to steady state mean of 124.06%), and plasminogen activity (increase from baseline mean of 116.38% to steady state mean of 150.13%). All other markers were changed in the opposite direction.

There were several statistically significant differences in change from baseline to days 1 and 10 of treatment in TPV/r subjects compared with DRV/r and RTV subjects (Table **4**). These differences were minor at day 1: a) aPTT larger mean decrease in the RTV group (-2.44 ± 2.29 seconds) than in the TPV/r group (-0.52 ± 1.48 seconds) and b) mean increase in Factor II in the DRV/r group (13.75 ± 16.8%) versus marginal mean decrease in the TPV/r group (2.90 ± 21.2%). Changes from baseline to day 1 in both of these factors in the TPV/r group were not statistically significant (Table **3**).

At day 10 there were more markers that demonstrated statistically significant differences between treatment groups. The increases in antithrombin III antigen, antithrombin III activity, and plasminogen activity were higher in the TPV/r group in comparison to the DRV/r group for the first marker. There were statistically significant differences between the TPV/r and RTV groups in fibrinogen and von Willebrand factor antigen. In addition, PAI-1 activity was increased to a lesser degree in the RTV group in comparison to the TPV/r group, and Factor X was increased to a lesser degree in the TPV/r group in comparison to the DRV/r group. With the exception of small increases in antithrombin III and plasminogen, mild increases in other studied factors with TPV/r suggests no increased risk for bleeding due to impeded coagulation or increase in fibrinolysis.

### Pharmacokinetic–Pharmacodynamic Evaluation

Regression analyses of TPV/r PK parameters (including log-transformed trough levels [*C*_min_] at all studied time points, *C*_max_ and AUC at steady state, and all tested PD biomarkers) did not provide for any consistent association between TPV/r PK parameters and effects on markers of platelet aggregation, fibrinolysis, and coagulation (data not shown). Higher plasma TPV trough concentrations were observed after 1 day of dosing (geometric mean 47.8 µM, CV% 46.9) compared with steady state (geometric mean 24.1 µM, CV% 80.5); however, there was no correlation between TPV trough plasma concentrations and inhibition of AA-induced platelet aggregation. In 6 out of 14 and 4 out of 7 subjects with *ex vivo* ≥ 50% inhibition of AA-induced platelet aggregation after 1 day and 10 days of TPV/r administration, respectively, TPV trough levels were consistently below the group mean. Conversely, 4 out of 7 subjects and 2 out of 9 subjects did not exhibit > 50% in *ex vivo* inhibition of AA-induced platelet aggregation after 1 day and 10 days of TPV/r administration, respectively, and showed consistently higher TVP trough levels above the group mean (not shown). Similarly, there was no correlation between studied PK parameters (*C*_max_, *C*_min_, and AUC_0-12_) and increases in CEPI CT with TPV/r treatment.

### Safety

Safety evaluations were performed on all enrolled subjects. All subjects in the DRV/r (*n* = 12) and RTV (*n* = 12) arms completed 10 days of treatment, whereas 70% (16/23) of subjects receiving TPV/r completed the 10-day treatment.

In the TPV/r arm, 70% of subjects (16/23) had at least 1 AE, including 14 subjects who had AEs that were considered treatment-related. A total of 7 subjects in TPV/r arm discontinued treatment early: 6 subjects due to AE and 1 subject because of non-compliance with the protocol (potential use of aspirin or other anti-platelet drugs during the washout period). Of 6 subjects who discontinued treatment early because of AEs, 5 subjects (2 of these were of severe intensity) had asymptomatic alanine aminotransferase (ALT) elevations (range, 89-334 U/l) that were > 2.5 times the upper limit of normal (ALT 6-34 U/l), that was pre-specified treatment discontinuation criterion, and 1 subject discontinued due to a severe pruritic rash. All 6 subjects with early discontinuation due to AEs completely recovered from these AEs during follow-up.

TPV trough concentrations prior to discontinuation in these subjects were substantially elevated (range, 54.3-197.5 µM) above the group mean (see above). In the other 2 PI arms, 50% (6/12) of subjects receiving DRV/r and 58% (7/12) receiving RTV had at least 1 AE and 3 and 5 subjects, respectively, had AEs defined as related to treatment. No subjects in the DRV/r or RTV groups discontinued because of AEs.

The most commonly reported AEs in the TPV/r, DRV/r, and RTV groups, respectively, included laboratory abnormalities (52%, 8%, 25%), gastrointestinal disorders (35%, 25%, 33%), nervous system disorders (13%, 42%, 17%), and skin disorders (9%, 17%, 25%). There were no serious AEs or deaths in this trial.

## DISCUSSION

Therapeutic doses of tipranavir in combination with ritonavir (TPV/r) at steady-state plasma concentrations significantly reduced *ex vivo* AA-induced platelet aggregation by a mean of 43% (AUC) in this randomized, controlled trial in healthy volunteers. This effect was more readily apparent after the first day of TPV/r administration when compared to steady state, but was not correlated with higher plasma TPV trough concentrations. The higher TPV concentrations on day 1 of treatment is explained by the immediate and potent inhibition of cytochrome P_450_ 3A4 enzyme by RTV, which was followed by later enzyme induction due to TPV [15]. Independent of TPV plasma concentrations, we observed large differences among subjects (e.g., inhibition of ≥ 50% versus no inhibition) on *ex vivo* AA-stimulated platelet aggregation, which may suggest differences in subject predisposition to TPV/r-mediated inhibition of AA-induced platelet aggregation. Similarly, and in addition to TPV/r effect, unknown factors may explain the abnormal PFA-100 CEPI CT tests in a minority of subjects who received TPV/r.

In our group of healthy volunteers, multiple dosing with TPV/r did not inhibit *ex vivo* collagen-, ADP-, and PFA-100 collagen/ADP-stimulated platelet aggregation function tests. These findings are in contrast to a recent study in which collagen- or ADP-stimulated platelet aggregation was inhibited in PRP from 5 HIV-infected patients taking TPV/r and in PRP taken from 5 healthy volunteers after it was spiked with different TPV concentrations [12]. In contrast to the *in vitro* experimental part of the study by Graff *et al*. [12], we observed a significant increase, and not a decrease, in TXB_2_ metabolite levels at TPV/r steady state, which persisted 48 hours following the last TPV/r dose. Differences in *in vivo* and *in vitro* study designs, research subjects, aggregometer, and reagents may account for some of the differences in the results. Further, Graff *et al*. [12] performed short time course experiments at 4 hours of *in vivo* dosing (results from 2 hours post-dosing indicated only non-statistically significant inhibition in collagen-stimulated platelet aggregation) or at about 1.5 hours of *in vitro* treatment allowing for evaluation of dynamic changes in platelet aggregation. Tests conducted in this study include both 1-day treatment changes (24 hours after the first dosing) and also reflect the steady state pre-dosing measurements. Nevertheless, the absence of an effect of TPV/r on collagen- and ADP-stimulated platelet function and the increase in urine TXB_2 _metabolites in our study are robust and are supported by a larger number of subjects (*n* = 21). Additionally, platelet viability and function was internally controlled for, as evidenced by the fact that several subjects had on-treatment abnormal AA-induced platelet aggregation and/or changes in PFA-100 CEPI CT, whereas concurrent collagen-, ADP- and PFA-100 collagen/ ADP-stimulated platelet aggregation function tests did not change from baseline.

Our results demonstrate that TPV/r specifically inhibits the pathway of AA-induced platelet aggregation in some, but not all subjects (e.g., in 43.8% for the primary endpoint at TPV/r steady state) while single-dose aspirin completely inhibited AA-induced platelet aggregation in all subjects not allowing for correlation analysis between aspirin and TPV/r effects. The mechanism by which TPV/r inhibits AA-induced platelet aggregation is different from that of aspirin. Our observation that a single dose of aspirin substantially reduced TXB_2_ metabolite levels in urine is consistent with its known inhibition of COX-1 [16] and is in contrast to the observed effect of TPV/r administration that resulted in elevated urine TXB_2_ metabolite levels. These findings are also consistent with a previous study in which 2 days of aspirin treatment reduced TXB_2_ serum levels in healthy volunteers by ~98% [14].

Same-subject comparison of treatment with a single dose of aspirin versus TPV/r showed that aspirin significantly inhibited other platelet functions, including PF-100 CEPI CT and collagen-stimulated platelet aggregation, providing for a much broader and more potent inhibition of different pathways of platelet aggregation when compared with TPV/r. Again, our observed effects of aspirin on AA-induced platelet aggregation and collagen-induced platelet aggregation are in agreement with those of previous studies in which aspirin [14] or COX-1 inhibitors [17,18] resulted in a profound (almost complete) inhibition of AA-induced platelet aggregation and inhibited collagen-induced platelet aggregation to a lesser extent (~50%) [14,18].

However, reversible TPV/r effects on *ex vivo *measures of platelet aggregation which we observed in some of our healthy volunteers should be interpreted with caution and may not translate into a real increase for the risk of bleeding events or ICH in HIV-infected patients receiving TPV/r-based cART and which are susceptible to TPV/r’s effect on platelet aggregation.

First, platelet aggregation is a complex process that includes several independent pathways, each of which can fully activate platelets leading to aggregation. Results from this study show that TPV/r significantly affected the pathway of AA-induced platelet aggregation in only some of the healthy volunteers, leaving other pathways of platelet aggregation intact. We show that TPV/r’s effect on AA-induced platelet aggregation is not aspirin-like and factors that may predispose subjects to the inhibition of AA-stimulated platelet aggregation by TPV/r are not known. We demonstrated that aspirin is a more potent inhibitor of AA-induced platelet aggregation and, consistent with previous observations, significantly inhibited several pathways of platelet aggregation.

Second, the tested HIV protease inhibitors led to small changes in markers of coagulation and fibrinolysis that were variable in their extent and direction. From the multitude of markers tested, overall we have found only AA-induced platelet aggregation to be consistently and substantially changed by TPV/r in some individuals. We did not find any clinically important effect of TPV/r treatment on a panel of coagulation factors (including aPTT and prothrombin time), markers of fibrinolysis and bleeding time that would suggest TPV/r causes deficiencies in coagulation factors or hemostasis mechanisms, including fibrinolysis, which could increase the risk of bleeding. Previous analysis of plasma samples from 87 patients from the RESIST trial that showed no difference in vitamin K-dependent factor levels in patients treated with TPV/r (*n* = 54) versus those receiving comparator PI/r (*n* = 30) [11] is consistent with our finding of absence of clinically important TPV/r effects on studied coagulation factors.

Third, the risk of bleeding, including ICH, is increased in individuals with HIV infection, and appears to be further increased in persons with AIDS [7,8,10,19-21]. This higher risk of bleeding occurs despite increased platelet activation [22], increased von Willebrand antigen levels [23], and the state of increased inflammation [24], collectively providing for a prothrombotic milieu in HIV infection [25]. Long-term suppressive antiretroviral therapy is associated with toxicities and does not completely reverse the inflammatory state and defects caused by HIV, which result in accelerated aging manifested by premature onset of cardiovascular disease, neurocognitive impairment, osteoporosis, and non–AIDS-related cancer [1,3,26-29]. The risk of ICH is increased with increased age, and patients on long-term antiretroviral therapy have an increased risk for ICH when compared with an age-matched population [9,10,30].

Finally, there is lack of clinical evidence that TPV/r increases the incidence of adverse bleeding or ICH. In clinical trials of TPV/r versus comparator PI/r, the incidence of adverse bleeding events (at any site) was 6.2 versus 5.1/100 person-years of exposure, respectively, with a risk ratio of 1.2 (95% CI, 0.78-1.80 (Boehringer Ingelheim: data on file). In prospective clinical trials, the incidence of ICH in TPV/r-treated patients was similar to that of HIV-infected patients in the pre-TPV era [9,10]. Exposure to any particular class of antiretroviral has not been found to increase the risk of ICH in a small but well-designed case-control study of 29 HIV-positive people with ICH and matched HIV-positive controls without ICH [20]. Eighty-eight percent of patients who developed an ICH in trials with TPV/r had AIDS. Furthermore, all patients who developed ICH in TPV/r trials had comorbid conditions such as CNS infection, cardiovascular disease, liver disease, or other conditions, and/or received concomitant anti-platelet or anti-coagulation therapy, all of which are known individual risk factors for ICH. Even though the platelet studies do not provide evidence for an increased risk for ICH that can be attributed to TPV/r therapy, TPV coadministered with low-dose RTV should be used with caution in patients who may be at risk of increased bleeding from trauma, surgery, or other medical conditions, or who are receiving medications known to increase the risk of bleeding, such as antiplatelet agents and anticoagulants, or who are taking high doses of vitamin E.

## Figures and Tables

**Fig. (1):**
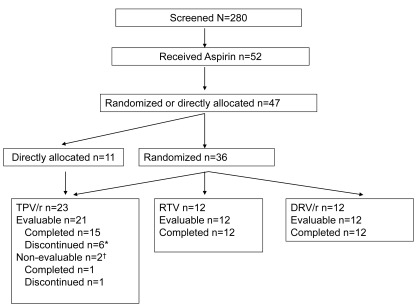
**Subject disposition.** *One subject missed only the last dose and the steady-state PK and PD data were considered valid, resulting in 16 evaluable subjects for steady-state analyses, but only 15 subjects for 24 and 48 hours post-dose analyses. ^†^Two subjects did not demonstrate return of platelet aggregation in response to AA after post-aspirin washout period.

**Fig. (2):**
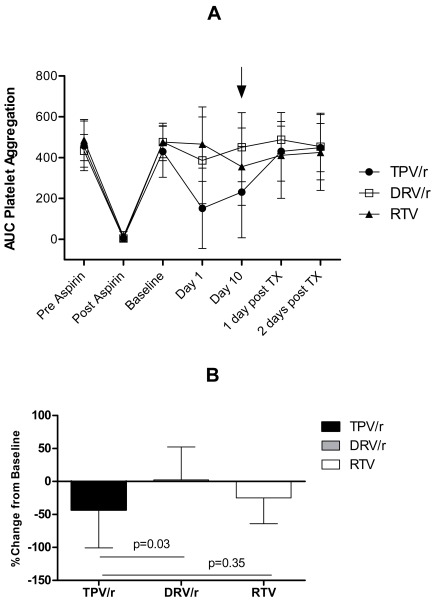
(**A**) Platelet aggregation in response to AA over time (mean AUC ± SD [AUC unit]); the arrow signifies steady state. (**B**) Comparison of platelet aggregation in response to AA at steady state between treatment groups. TX = treatment.

**Fig. (4):**
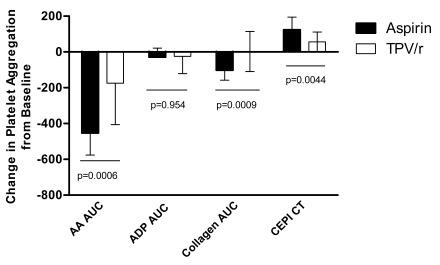
Within-subject comparison of aspirin and TPV/r (at steady state) on platelet aggregation.

**Table 1:** Patient Demographics and Baseline Characteristics

	TPV/r	DRV/r	RTV	Total
	*n* = 23	*n* = 12	*n* = 12	*N* = 47

Gender, N (%)				
Men	14 (60.9)	7 (58.3)	7 (58.3)	28 (59.6)
Women	9 (39.1)	5 (41.7)	5 (41.7)	19 (40.4)

Ethnicity, N (%)				
Not Hispanic or Latino	20 (87.0)	11 (91.9)	10 (83.3)	41 (87.2)
Hispanic or Latino	3 (13.0)	1 (8.3)	2 (16.7)	6 (12.8)

Mixed race, N (%)				
Yes	1 (4.3)	0	0	1 (2.1)
No	22 (95.7)	12 (100)	12 (100)	46 (91.9)

Race, N (%)				
White	5 (21.7)	1 (8.3)	4 (33.3)	10 (21.3)
Black	17 (73.9)	11 (91.7)	7 (58.3)	35 (74.5)
Asian	1 (4.3)	0	1 (8.3)	2 (4.3)

Mean age, y	32.9	34.0	31.8	32.9

Mean body mass index kg/m^2^, % ≥ 30 (minimum-maximum)	27.7/22%	27.8/25%	26.5/25%	27.4/23%
	(21.5-36.3)	(22.4-40.2)	(20.5-30.6)	(20.5-40.2)

Smoking history, N (%)				
Never smoked	19 (82.6)	10 (83.3)	10 (83.3)	34 (72.3)
Ex-smoker	4 (17.4)	2 (16.7)	2 (16.7)	13 (27.7)
Current smoker	0	0	0	0

Alcohol history, N (%)				
Non-drinker	14 (60.0)	10 (83.3)	10 (83.3)	34 (72.3)
Average consumption	9 (39.1)	2 (16.7)	2 (16.7)	13 (27.7)
Excessive consumption	0	0	0	0

**Inclusion criteria:** Healthy men and women aged 18-50 years who agreed to abstain from alcohol, recreational drugs, tobacco products, ingestion of grapefruit juice or Seville oranges during the study; were negative for HIV-1 and hepatitis C antibody, or hepatitis B surface antigen; and had normal platelet count ≥ 125,000/mm^3^, hemoglobin ≥ 11.0 g/dl, prothrombin time ≤ 1 x upper limit of normal (ULN), and activated partial thromboplastin time ≤ 1 x ULN.

**Table 2:** Course and Change from Baseline for Other Platelet Functions in the TPV/r Group

Biomarker (Reference Range)		Baseline	Day 1	Day 10	24 h After Last Dose	48 h After Last Dose
		*n* = 21	*n* = 21	*n* = 16	*n* = 15	*n* = 15

Bleeding time, min (< 8 min)	Mean	5.29	6.48	5.47	5.73	4.67
	p*	–	0.056	0.694	0.410	0.104

Urinary TxB_2_ metabolites, pg/mg (> 1500)	Mean	2748	2458	3609	3374	4272
	p*	–	0.123	0.019	0.116	0.027

PFA-100 collagen/epinephrine, s (94-193)	Mean	101.1	193.1	161.7	118.9	108.8
	p*	–	<0.0001	0.0011	0.097	0.756

PFA-100 collagen/ADP, s (71-118)	Mean	70.62	73.81	74.38	74.33	70.53
	p*	–	0.366	0.619	0.864	0.110

* Paired *t*-test compared with baseline.

**Table 3:** Course and Change from Baseline in Biomarkers of Coagulation and Fibrinolysis With TPV/r

Biomarker (Reference Range)		Baseline	Day 1	Day 10	24 h After Last Dose	48 h After Last Dose
		*n* = 21	*n* = 21	*n* = 16	*n* = 15	*n* = 15

aPTT, s (22.8-31.0)	Mean	24.9	24.37	22.46	22.01	22.19
	P*		0.122	<0.0001	<0.0001	0.0002

Prothrombin time, s (9.7-12.3)	Mean	10.37	10.33	10.09	9.99	9.98
	P*		0.438	0.019	0.0011	0.0009

Fibrinogen, mg/dl (170-330)	Mean	373.52	385.05	431.63	458.6	469.0
	P*		0.476	0.0031	0.0004	0.006

von Willebrand Factor antigen, % (> 49)	Mean	153.71	166.62	196.94	197.07	170.0
	P*		0.187	0.035	0.0085	0.0325

Antithrombin III antigen, % (80-124)	Mean	104.29	108.75	134.44	131.0	130.93
	P*		0.107	<0.0001	<0.0001	<0.0001

Antithrombin III activity, % (80-120)	Mean	100.57	103.24	124.06	120.53	115.87
	P*		0.062	<0.0001	<0.0001	<0.0001

Factor II, % (50-200)	Mean	110.38	107.48	129.19	122.40	121.27
	P*		0.538	<0.0001	0.0753	0.0191

Factor VII, % (50-200)	Mean	106.81	103.81	135.38	135.00	122.73
	P*		0.619	<0.0001	0.0041	0.0862

Factor IX, % (50-200)	Mean	87.29	81.86	99.56	102.13	104.87
	P*		0.240	0.044	0.066	0.0147

Factor X, % (50-200)	Mean	112.10	110.57	124.94	133.47	125.33
	P*		0.743	0.0006	0.0012	0.0164

Plasminogen activity, %	Mean	116.38	119.9	150.13	145.4	142.93
	P*		0.009	<0.0001	0.002	0.0073

α-2 antiplasmin activity, %	Mean	105.81	106.76	108.5	109.67	105.93
	P*		0.351	0.169	0.187	0.669

D-dimer, mg/dl	Mean	1.7	1.78	1.42	1.36	1.34
	P*		0.098	0.067	0.087	0.115

PAI activity, IU/ml	Mean	8.29	14.19	21.44	17.33	16
	P*		0.014	0.0003	0.021	0.008

* Paired t-test compared with baseline.

**Table 4:** Comparison of Changes in Platelet Function,* Coagulation, and Fibrinolytic Biomarkers by Treatment†

		Mean Change from Baseline (SD)	
		Day 1	Day 10
Urinary TxB_2_ metabolites, pg/ml	TPV/r	-290(804)	955 (1458)
	DRV/r	-31.42 (651)	232.6 (772)
	RTV	-671(1799)	-456.21 (1473)‡
PFA-100 CEPI CT, s	TPV/r	92.05 (73.4)	56.06 (55.4)
	DRV/r	-3.92 (15.2)‡	0.583 (14.0)‡
	RTV	-11.17 (19.9)‡	-6.58 (21.6)‡
aPTT, s	TPV/r	-0.52 (1.48)	-2.79 (1.75)
	DRV/r	-0.70 (2.22)	-1.5 (1.81)
	RTV	-2.44 (2.29)‡	-2.53 (3.78)
Fibrinogen, mg/dl	TPV/r	11.52 (72.8)	66.93 (76.1)
	DRV/r	33.16 (40.3)	46.91 (120)
	RTV	-40.25 (94.2)	-31.33 (118)‡
Von Willebrand factor antigen, %	TPV/r	12.90 (43.2)	46.5 (80.3)
	DRV/r	17.5 (20.9)	28.08 (64.8)
	RTV	-17.25 (48/6)	-13.3 (50.0)‡
Anti-thrombin III activity, %	TPV/r	2.66 (6.19)	25.5 (15.7)
	DRV/r	18.66 (35.7)	13.5 (14.3)‡
	RTV	6.00 (29.6)	10.16 (13.3)‡
Anti-thrombin III antigen, %	TPV/r	4.28 (11.6)	30.75 (14.4)
	DRV/r	6.25 (8.48)	19.58 (6.89)‡
	RTV	0.41 (11.1)	22.58 (10.8)
Factor II, %	TPV/r	-2.90 (21.2)	19.5 (13.6)
	DRV/r	13.75 (16.8)‡	20.5 (16.2)
	RTV	-3.19 (9.42)	8.5 (14.8)
Factor X, %	TPV/r	-1.52 (21.0)	11.43 (10.6)
	DRV/r	6.91 (15.6)	26.16 (13.7)‡
	RTV	-4.08 (14.2)	5.08 (14.0)
Plasminogen activity, %	TPV/r	3.52 (5.6)	31.6 (23.2)
	DRV/r	7.58 (11.2)	14.8 (16.2)‡
	RTV	4.75 (8.6)	21.0 (14.6)
PAI-1 activity (IU/ml)	TPV/r	5.90 (10.0)	11.5 (9.8)
	DRV/r	2.25 (4.6)	6.83 (9.3)
	RTV	1.58 (6.3)	2.75 (10.2)‡

* Excludes LTA tests.

† Presented markers are limited to those that had at least one p value > 0.05.

‡ p<0.05; p-values represent 2-sided *t*-test for any difference in mean change from baseline of TPV/r and DRV/r or RTV.
